# Sudden deaths due to accidental leakage of Lindane from a storage tank in a village, Sitapur, Uttar Pradesh, India, 2020: A field epidemiological investigation

**DOI:** 10.1097/EE9.0000000000000213

**Published:** 2022-06-08

**Authors:** Piyush Jain, Amit Kapoor, Polani Rubeshkumar, Mohankumar Raju, Bency Joseph, Prashant Bhat, Parasuraman Ganeshkumar, Chandrasekharan Nair Kesavachandran, Devendra Kumar Patel, Natesan Manickam, Prabhdeep Kaur

**Affiliations:** a ICMR-National Institute of Epidemiology, Chennai, India; b Department of Health and Family Welfare, Uttar Pradesh, India; c CSIR-Indian Institute of Toxicology Research, Lucknow, Uttar Pradesh, India

**Keywords:** Chemical accidents, Hazardous chemicals, India, Lindane

## Abstract

**Background:**

Chemical leakages cause devastating health effects on humans. On 6 February 2020, seven deaths were reported following a hazardous chemical leakage in a village in Uttar Pradesh, India. We investigated the event to identify the cause and propose recommendations.

**Methods:**

We defined a case as sudden onset of breathlessness, headache, or death in the village, 6–7 February 2020. We conducted a house-to-house case search and calculated attack rate (AR) and case-fatality rate (CFR) by age and gender. We conducted an environmental investigation at the leakage site and sent the chemicals for forensic analysis. We obtained the cause of death through autopsy reports.

**Results:**

Out of 2,942 residents, we identified 23 cases (AR = 8/1,000) and seven deaths (CFR = 30%). The median age of the case was 42 years (range, 2–64 years). The AR was higher among males (14/1,000 [19/1,402]). All the 23 case-patients who were sleeping at the chemical leakage site or visited to witness the event developed symptoms, and all seven cases who were sleeping within 150 meters of the leakage site died. The environmental investigation revealed leakage of hazardous substances from the storage tank. Toxicology analysis confirmed the leaked chemical as Lindane (gamma-hexachlorocyclohexane), and autopsy reports confirmed the cause of death as asphyxia.

**Conclusions:**

Asphyxia following the leakage of Lindane from the storage tank possibly led to sudden deaths. We recommend using leak-proof tanks to ensure safe storage and disposal, law enforcement, and regulations to prevent people from staying close to chemical storage sites.

**What this study adds?**

Technological disasters such as chemical leaks are rarely documented in the scientific literature. We investigated leakage of Lindane (gamma-hexachlorocyclohexane) in a village in India using the field epidemiology methods to investigate outbreak due to suspected chemical etiology. This study documents the adverse health events following accidental leakage of Lindane due to improper storage protocols and importance of emphasizing following regulations.

## Introduction

Disasters are categorized into two main groups: natural and technological disasters.^1^ A technological disaster is an event caused by a malfunction of a technological structure and/or some human error in controlling or handling the technology.^1,2^ Chemical spill, explosion, oil leak, gas leak, poisoning, radiation, and deep-water horizon constitutes the technological disasters.^1,2^ The technological disasters cause health effects such as stress, disabilities, and sometimes deaths.^1,2^ World Health Organization estimated 65,000 deaths globally due to technological disasters between 2009 and 2018.^3^ One in three (36%) disasters are technological, however, only major events such as Bhopal gas leak and Chernobyl radiation leak are reported. Among the technological disasters, 70% of the disasters are transport-related and 16% industries-related.^2^

India reported 356 technological disasters during 2000–2019.^2^ In 1984, Bhopal gas tragedy due to leakage of methyl isocyanate gas led to >3,000 deaths and adverse health effects for a large population.^4^ Recently, 12 died and hundreds injured following styrene gas leak in Visakhapatnam on May 2020.^5^ Reasons for such incidents are poor compliance to safety protocols and deviation from specified procedures.^6^ On 6 February 2020, the media reported sudden deaths following an unknown hazardous gas leak in a village, Sitapur district, Uttar Pradesh, India. The village has around 35 industries and approximately 610 households, mostly from low-income group. State Disaster Response Force (SDRF) conducted rescue operations as per the protocol. A team of epidemiologist, physician, scientists with expertise in environmental sciences and toxicology conducted a technical investigation with the objectives; to describe the events that led to sudden deaths, identify the cause, and propose recommendations.

## Methods

The team along with frontline workers initiated the investigation on 7 February 2020. We adopted the field epidemiology method to investigate the outbreak due to suspected chemical etiology.^7–9^ We coined a case definition based on the symptoms reported by the cases and clinical examination by physicians. We defined a case as sudden onset of breathlessness, headache, nausea, or death in any resident of the village, 6–7 February 2020. A team of frontline workers conducted an active house-to-house case search in all the households of the village and line-listed the cases meeting the case definition. We collected data on demographics, clinical symptoms, and exposures through face-to-face interview. A physician examined symptomatic individuals and collected information about the deceased from their family members. We interviewed a few key informants including cases and village residents about the events to generate hypotheses. We described the clinical symptoms and calculated the attack rate and case-fatality rate by age and gender. We constructed a spot map to describe the relationship between the cases and the chemical leakage site.

### Environmental and forensic investigation

A team of experts inspected the chemical leakage site and searched for animal and bird deaths in the vicinity. They collected water samples according to United States Environmental Protection Agency Wastewater Sampling (306) AF.R4 method from the three locations near to the leakage sites without using automatic sampler.^10^ The glass bottles were properly rinsed and the water sample were filled without having any headspace. Soil samples from the leakage site were collected according to test methods for evaluating solid waste using physical/chemical methods (SW-846) and method 5035 for Volatile Organic Compounds (VOCs).^11^ The soil samples were collected using manual handheld scoops and kept in plastic bags. Samples were transported to laboratory within 3 hours and stored at 4 °C till extraction. The extracted samples were analyzed for pesticides residue on gas chromatography-mass spectrometry, Thermo TSQ Quantum XLS instrument for Lindane (Thermo Fisher Scientific, Waltham, MA) and 53 other pesticides residues. The level of pesticide residues was assessed against the below quantification limit (BQL) (detection limit = 0.1 ηg/ml) in the collected samples.

A forensic medicine expert performed the autopsy of the deceased case-patients in the district hospital.

## Results

We identified 23 cases among the 2,942 residents of the village. The median age of the case-patient was 42 years (range, 2–64 years). The overall attack rate of sudden onset of symptoms was eight per 1,000 population. The attack rate was higher among males (13/1,000 population) than females (3/1,000 population) (Table 1). Among the 23 cases, seven were deceased; the case-fatality rate was 30%. Among the deceased, five were males and three were <15 years old. Of 16 alive case-patients, 10 (65%) reported breathlessness, eight (52%) had headache and nausea, and seven (43%) reported metallic taste. Among the 16 survivors, 14 were treated as outpatient and two were hospitalized in a public health facility. Both were discharged after 24 hours of observation.

**Table 1.** Attack rate among the resident of the village, Sitapur, Uttar Pradesh, India, 6–7 February 2020

Characteristics	No. cases	Population at risk	Attack rate per 1,000 population
Age (years)			
1–10	2	346	6
11–20	2	410	5
21–30	2	478	4
31–40	4	507	8
41–50	7	780	9
51–60	4	203	20
61–70	2	218	9
Gender			
Male	19	1,402	14
Female	4	1,540	3
Total	23	2,942	8

### Findings of the environmental and forensic investigations

**Figure 1.**
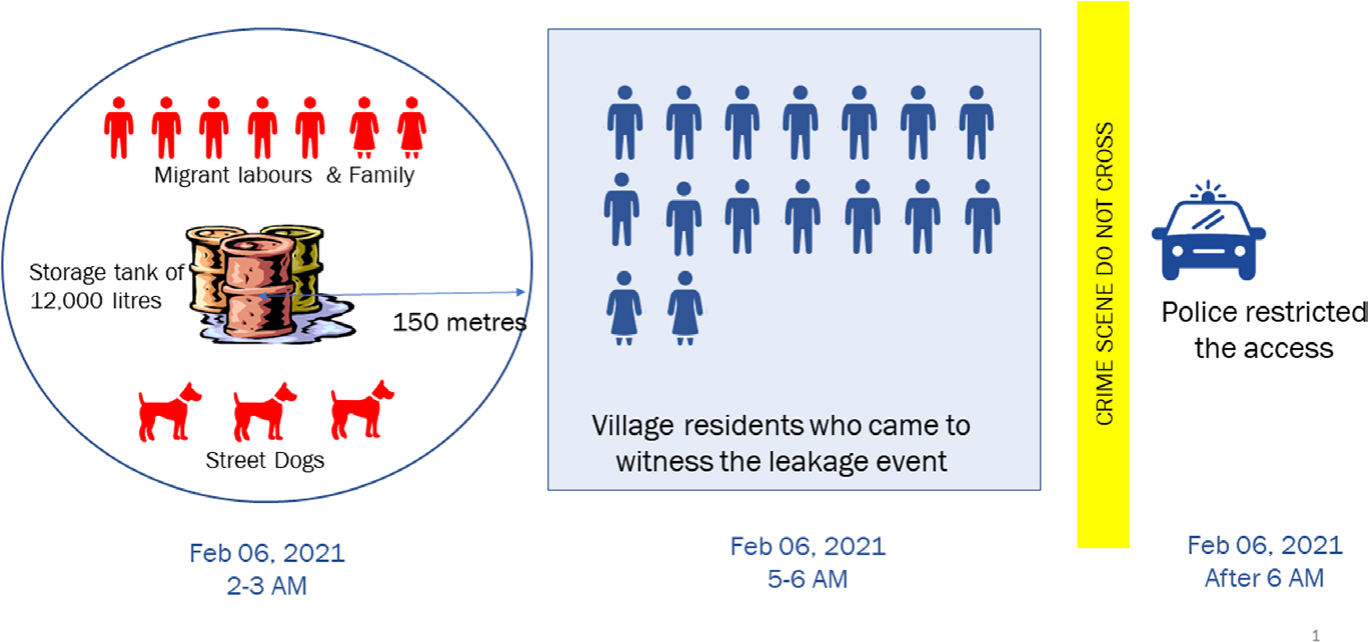
Description of chemical leakage site and sequence of events led to sudden deaths of migrants, Sitapur, Uttar Pradesh, India, 2020 (n = 23).

Industries stored the chemical waste in a temporary tank with a storage capacity of 12,000 liters. Migrant laborers with their families lived in temporary settlements within a radius of 150 to 200 meters of the leakage site. The storage tank was located approximately 1.5 miles (2.4 kilometers) away from the closest residential area. Our investigation indicated that chemicals leaked from the storage tank, possibly between 2 am and 3 am (Indian standard time), in the early morning hours of 6 February 2020. Seven individuals, including three children, and three street dogs, were found dead within the vicinity of 150–200 meters of the leakage site (Figure 1). Between 5 am and 6 am, 6 February 2020, 16 residents of the village visited the incident area. All of them developed symptoms rapidly within 2 hours of the exposure, and no deaths were reported among them. By 6 am, 6 February 2020, the Police force barricaded the area and restricted entry to the leakage site. Neither cases nor animal deaths were reported in the nearby villages. Despite our efforts, we could not confirm the cause of the leakage.

The toxicological analysis revealed the excess quantities of lindane (gamma-hexachlorocyclohexane) in water (0.461–0.500 μg/L) and soil (1.030–2.100 μg/L). The level of other pesticide residues was within permissible limits in water and soil. Autopsy reports of all seven deceased individuals confirmed the cause of death as asphyxia.

The team explored the reason for the death of seven individuals while the others recovered the next day. All those who got exposed to the chemical leak (100% [23/23]) within the first 4 hours of the accident (i.e., 2 am to 6 am) developed symptoms. All seven dead individuals stayed overnight within the 150-meter radius around the leakage site.

## Discussion

The epidemiological and toxicological analysis suggested that sudden deaths on 6 February 2020, in the village were possibly due to Lindane exposure. Lindane, is also known as gamma-hexachlorocyclohexane, is an organochlorine class of pesticides.^12^ It is used as an insecticide for crops and a second-line drug for scabies and head lice.^12^ Many countries banned the use of Lindane following the Stockholm convention on persistent organic pollution.^13^ Lindane has been banned for manufacture, import, and use in India since 2013.^14^ Maximum permissible level of Lindane in water is 0.0002 mg/L.^15^

In our investigation, we found residual Lindane in liquid form stored in the storage tank. Lindane is volatile and more toxic at a lower temperature.^16^ Due to its volatile nature, it possibly turned to gas form on leakage from the storage tank. The toxicological analysis estimated twice the permissible levels of Lindane in the water near the leakage site. All seven migrants and three dogs in the proximity of the tank might have been exposed to the Lindane. Lindane overdose can cause respiratory depression.^16^ The deceased possibly developed respiratory depression and died eventually due to asphyxia, which was in agreement with the autopsy results of the deceases.^16,17^ Restricted access to leakage site by barricading could have prevented the other village residents from developing the symptoms.

The most common reasons for technological disasters are negligence, breaching the regulations, and an untrained workforce.^2,6^ The policy frameworks such as National Disasters Management Guidelines-Chemical disasters^6^ and manufacture, storage, and import of hazardous chemicals, 1989,^18^ describes protocols for responding to emergencies, safe storage, and periodic risk assessment. These guidelines should be implemented strictly to avoid similar incidents in the future. We established an epidemiological linkage between the exposure (Lindane) and cases (deaths) using standard investigation methodology.^7–9^ The findings of the environmental and forensic analysis reinforced the same. Furthermore, the clinical effects of Lindane poisoning were consistent with the published literature. Our investigation had a major limitation that we did not quantify the concentration of Lindane in the body fluids. Uttar Pradesh is one of the States with low per capita (US$ 991) and the accident took place in a remote location with limited resources.^19^ Due to lack of facilities, quantification of the chemicals was not feasible.

We conclude that leakage of Lindane from the chemical storage tank possibly led to the sudden deaths. We recommended using leak-proof tanks to store chemical waste, ensure safe disposal of the chemical wastes as per the standards and regulations, enforcement of existing laws, and restricting residential areas near chemical storage sites.

## Acknowledgments

We acknowledge Vipin Bihari (late) and Alok Dhawan, CSIR-Indian Institute of Toxicology Research, Lucknow, India, for their field support and facilitating the investigation.

## Conflicts of interest statement

The authors declare that they have no conflicts of interest with regard to the content of this report.

## References

[1] Lindsey A Donovan M Smith S Radunovich H Gutter M. *FCS9265/FY1230: Impacts of Technological Disasters*. 2021. Available at: https://edis.ifas.ufl.edu/publication/FY1230. Accessed 19 October 2021.

[2] Centre for Research on the Epidemiology of Disasters. CRED Crunch Issue 60. *Technological Disasters*. UCLouvain. 2020. Available at: https://cred.be/sites/default/files/CC60.pdf. Accessed 19 October 2021.

[3] World Health Organization. Chemical Incidents. 2020. Available at: https://www.who.int/westernpacific/health-topics/chemical-incidents. Accessed 23 August 2021.

[4] Dhara VR Dhara R. The Union Carbide disaster in Bhopal: a review of health effects. Arch Environ Health. 2002;57:391–404. 12641179 10.1080/00039890209601427

[5] Dhara VR Digumarti R Sridhar GR Gassert TH. The styrene gas disaster – lessons to learn and the way forward. J Dr NTR Univ of Health Sci. 2021;10:117.

[6] National Disaster Management Authority, Government of India. Chemical Disasters. 2007:124. Available at: https://nidm.gov.in/pdf/guidelines/new/chemicaldisaster.pdf. Accessed 17 April 2022.

[7] Centers for Disease Control and Prevention. *Principles of Epidemiology: Lesson 6, Section 2|Self-Study Course SS1978|CDC*. 2021. Available at: https://www.cdc.gov/csels/dsepd/ss1978/lesson6/section2.html. Accessed 23 February 2022.

[8] Murhekar M Moolenaar R Hutin Y Broome C. Investigating outbreaks: practical guidance in the Indian scenario. Natl Med J India. 2009;22:252–256. 20334049

[9] World Health Organization. Manual for Investigating Suspected Outbreaks of Illnesses of Possible Chemical Etiology: Guidance for Investigation and Control. 2021. Available at: https://www.who.int/publications/i/item/9789240021754. Accessed 17 April 2022.

[10] United States Environment Protection Agency. *Procedures for Collecting Wastewater Samples*. 2015. Available at: https://www.epa.gov/quality/procedures-collecting-wastewater-samples. Accessed 17 April 2022.

[11] United States Environment Protection Agency. *SW-846 Update VII Announcements*. 2018. Available at: https://www.epa.gov/hw-sw846/sw-846-update-vii-announcements. Accessed 17 April 2022.

[12] Nantel AJ. *LINDANE (PIM 859*). 2001. Available at: http://www.inchem.org/documents/pims/chemical/pim859.htm. Accessed 19 August 2021.

[13] Secretariat of Stockholm Convention. *Lindane*. 2007. Available at: http://chm.pops.int/Implementation/%20Alternatives/%20AlternativestoPOPs/%20ChemicalslistedinAnnexA/%20Lindane/tabid/5865/Default.aspx. Accessed 23 February 2022.

[14] ICAR-Indian Institute of Spices Research. Pesticides/Formulations Banned in India. 2018. Available at: http://www.spices.res.in/pages/pesticides-formulations-banned-india. Accessed 23 February 2022.

[15] National Toxicology Program. Lindane. U.S. Department of Health and Human Services. 2009. Available at: https://ntp.niehs.nih.gov/ntp/roc/content/profiles/lindane.pdf. Accessed 6 March 2022.

[16] California Poison Control System. Lindane and Organochlorine Poisoning. 2010. Available at: https://calpoison.org/news/lindane-organochlorine-poisoning. Accessed 19 August 2021.

[17] Oregon State University. Extension Toxicology Network Pesticide Information Profiles - LINDANE. 1996. Available at: http://extoxnet.orst.edu/pips/lindane.htm. Accessed 24 August 2021.

[18] Ministry of Environment and Forests, Government of India. The Manufacture, Storage, and Import of Hazardous Chemicals Rules, 1989. 1989:58. Available at: http://nagarikmancha.org/images/MANUFACTURE,%20STORAGE%20AND%20IMPORT%20OF%20HAZARDOUS%20CHEMICAL%20RULES,%201989.pdf. Accessed 23 February 2022.

[19] Reserve Bank of India. Handbook of Statistics on Indian States. 2021. Available at: https://m.rbi.org.in/scripts/AnnualPublications.aspx?head=Handbook+of+Statistics+on+Indian+States. Accessed 17 April 2022.

